# Patient outcomes after attending pre-pump education class: disparities in initiation and glycemic outcomes

**DOI:** 10.3389/fendo.2025.1568133

**Published:** 2025-06-05

**Authors:** Marinés Castillo Echevarría, Riley E. Evans, Jessica A. Schmitt

**Affiliations:** ^1^ Department of Pediatrics, University of Alabama at Birmingham Heersink School of Medicine, Birmingham, AL, United States; ^2^ University of Alabama at Birmingham Heersink School of Medicine, Birmingham, AL, United States

**Keywords:** diabetes mellitus, type 1, adolescent, child, artificial pancreas, closed-loop systems

## Abstract

**Background:**

At Children’s of Alabama, all patients with insulin dependent diabetes are considered for continuous subcutaneous insulin infusion therapy (CSII, also known as insulin pump). In some cases, eligibility depends on insurance requirements, including six months of MDI therapy, CSII education, and a six-week glucose log with four daily readings.

**Objective:**

Evaluate factors influencing CSII initiation after “Prepump class” and assess glycemic changes in CSII starters.

**Methods:**

A retrospective review of pre-pump class attendees from January 2022 to July 2023 was completed. Patients who initiated a CSII prior to January 2024 were identified as “CSII-starters,” and those who remained in multiple daily injections were identified as “MDI-retainers”. Demographic and medical data were compared between these groups. For CSII-starters, type of system and use of automatic insulin delivery (AID) was evaluated. Glycemic outcomes were assessed in those with type 1 diabetes (T1D) with continuous glucose monitor data. A sub-analysis was done for those outside the honeymoon period. Outcomes of AID systems and users of non-AID systems were compared.

**Results:**

Of 283 pediatric patients who attended pre-pump class, 187 (66%) started CSII, with a median initiation time of 108 days (interquartile range 76–154). CSII-starters and MDI-retainers differed by race (p=0.0385) and insurance (p=0.0001), but not by sex, language, or age at diagnosis. Initiators were younger (p=0.0150), had shorter diabetes duration (p=0.0001), lower HbA1c (p=0.0020), and higher CGM use (p<0.0001). Among starters, 70% chose tubeless pumps, and 62% (n=116) used AID systems. Race and insurance were not linked to AID vs non-AID choice, but were associated with CSII initiation. Insurance and race were not associated with selecting an AID over non-AID CSII systems. In glycemic analysis, 183 patients were studied. AID systems showed improved outcomes compared to non-AID systems for the full analysis and sub-analysis of patients outside the honeymoon period.

**Conclusion:**

While race and insurance are not associated with the selection of an AID vs non-AID system, they are associated with CSII-starters versus MDI-retainers. As expected, AID systems outperformed non-AID systems in our cohort. Future work will aim to reduce disparities in CSII and AID access for all interested in diabetes technology.

## 1 Introduction

Type 1 diabetes mellitus (T1D), caused by insulin deficiency following autoimmune destruction of the pancreatic beta cells, is the most common form of diabetes in youth (1, 2). Aggressive management with frequent glucose monitoring and insulin administration is associated with a reduced risk of complications (3–5).

Insulin can be given via multiple daily injections (MDI) or continuous subcutaneous insulin infusion (CSII, also known as an insulin pump). Relative to MDI, CSII use is associated with improved glycemic control (6–8) and lower hospitalization rates for diabetes-related emergencies (9, 10). Therefore, CSII should be considered a viable treatment option for pediatric patients with diabetes, particularly those with suboptimal glycemic control, as it has the potential to improve both therapeutic efficacy and overall safety in this population (11, 12).

With technological advances in CSII and continuous glucose monitors (CGM), the available CSII options have evolved. Currently, options include tubeless and tubed CSII models as well as CSII systems with or without automatic insulin delivery (AID) (13). An AID system allows for automatic insulin administration of basal insulin in addition to correction boluses dependent on real time CGM measurements (7, 14). The use of a CSII with AID is the current standard of care in patients able to safely use the technology (11). This combination is strongly recommended for school-aged children and adolescents, and should also be considered for use in toddlers and preschool-aged children as well as those with hypoglycemia unawareness (12).

While this is the standard of care, there are multiple factors that limit access and use of CSII therapy in general and AID systems for many patients. Patient CGM preference, provider bias, and insurance requirement can limit access. This variation raises concerns for unequitable access, something that has been seen with diabetes-technologies in the past (15).

At Children’s of Alabama, anyone is eligible for CSII therapy if the caregiver, provider, and the patient mutually agree to pursue it. However, eligibility is also subject to insurance coverage limitations. For example, Alabama Medicaid requires six months of MDI therapy, education on CSII, and a documented six-week blood glucose log with four discrete values per day. CGM reports do not count as discrete values. At Children’s of Alabama, an in-person pre-pump class is offered by Certified Diabetes Care and Education Specialists (CDCES) to patients with diabetes mellitus who are interested in starting a CSII. During this 3-hour class, which fulfills Medicaid education requirements, a group of families and patients receive education on the differences between CSII and MDI, the different features of all available CSII models, and a comparison of AID and non-AID systems. This class does not favor one CSII over the others, and families have access to all the devices that are available and approved for children. While this formal education is required by Alabama Medicaid, it is optional for most other insurers.

**Figure 1:**
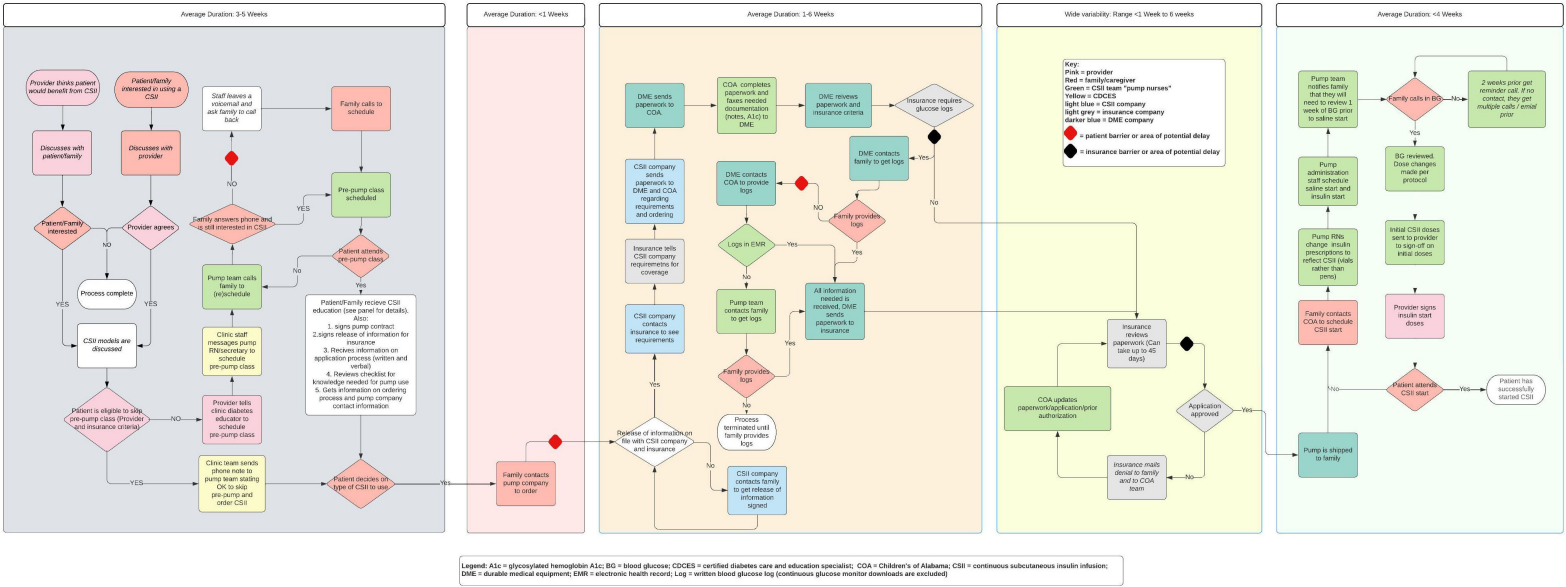
Process map of CSII initiation at Children’s of Alabama circa 2023. BG, blood glucose; COA, Children’s of Alabama; CSII, continuous subcutaneous insulin infusion; DME, durable medical equipment; EMR, electronic medical record.

In our system, significant steps are required to initiate a CSII (See **Figure 1**), and the process requires cooperation between the clinic, durable medical equipment providers or pharmacy, the insurer, and the patient’s family. In this complex process, we were unsure what proportion of patients who attended pre-pump class were successfully able to start a CSII system and if there were any significant demographic or medical differences between those able to start a CSII (CSII-starters) compared to those who remained on MDI (MDI-retainers) or if there were any demographic or medical differences in those who selected a CSII with an AID or non-AID capacity.

While the primary goal was to evaluate the outcomes of attendance to pre-pump class on CSII initiation and selection, a secondary goal was to evaluate glycemic outcomes for a subset of patients with T1D who transitioned from MDI to CSII.

## 2 Materials and methods

### 2.1 Objectives

*Primary Aim:*

1. Assess outcomes of existing process as related to access to CSII therapy among pediatric patients with insulin dependent diabetes who attended a “pre-pump” class.

*Objectives:*

1. Evaluate the patients who attended a pre-pump class and explore factors that are associated with CSII initiation.
2. Evaluate the features of CSII selected and evaluate differences in those selecting AID compared to non-AID models.
3. Assess glycemic outcomes, measured by hemoglobin A1C (HbA1c) and CGM metrics, in pediatric patients with T1D who transitioned from MDI therapy to CSII therapy.
4. Conduct a sub-analysis of glycemic outcomes in youth with T1D who had been diagnosed for more than 12 months prior to CSII initiation, and who had 14-day continuous glucose monitoring (CGM) data available both before and after starting CSII, in order to minimize the influence of the “partial remission” or “honeymoon” phase.

### 2.2 Participants

This retrospective study included patients with diabetes seen from January 2022 through December 2023 at Children’s of Alabama. Patients below 21 years of age who attended pre-pump class from January 2022 through June 2023. Patients who had documented CSII use by December 2023 were defined as “CSII-starters.” Patients without documented CSII use by December 2023 were described as “MDI-retainers.” The six-month phase from July 2023 through December 2023 was included to allow for time for those attending pre-pump class in June 2023 to have adequate time to decide upon CSII therapy, navigate the complex process (See **Figure 1**), and start their CSII. Patients above 21 years of age and those who attended a pre-pump class outside the observation period from January 2022 through December 2023 were excluded from the patient cohort. Type of diabetes was not an exclusion criterion, for the primary and secondary aim. Glycemic evaluation was limited to those with T1D transitioning from MDI to CSII with sub-analysis limited as noted above to account for confounding of the honeymoon phase.

### 2.3 Data source

The patient cohort was identified from pre-pump class attendance records kept by the CDCES team leading the pre-pump class. Demographic information (age, legal sex, language, race/ethnicity, and insurance), clinical data (age at diagnosis of diabetes, duration of diabetes at pre-pump class, duration of diabetes at CSII initiation, and HbA1c), and diabetes-technology information (date of the CSII initiation, CSII model, and if model was used in AID mode) were retrieved from the electronic health record via individual chart review.

The CGM metrics were obtained directly from patient’s shared data with the clinic. “Pre-CSII HbA1c” was the last recorded HbA1c prior to CSII initiation and “post-CSII HbA1c” was that closest to 90 days after CSII initiation. CGM metrics were collected for the 14-days prior to HbA1c measurement. For patients without a post-CSII HbA1c measurement but with available CGM data, a 14-day CGM data window was selected for analysis. The end date of this window matched the median number of days post-CSII initiation when HbA1c measurements were obtained in the cohort. For example, if the cohort’s median timing for post-CSII HbA1c measurement was 120 days after CSII initiation, CGM data from days 106 to 120 post-CSII would be analyzed for these patients.

### 2.4 Variables

Language was identified as English and non-English speaking. The race/ethnicity was defined as non-Hispanic White (NHW), non-Hispanic Black (NHB), Hispanic and other. Insurance was subdivided to Medicaid and non-Medicaid insurance.

Commercially available CSII models during the study period that were selected by CSII-starters were: Omnipod^®^ UST400, Omnipod^®^ DASH, Omnipod^®^ 5, Medtronic MiniMed™ 770 G, Tandem Control IQ^®^, and Tandem Basal IQ^®^. The CSII models were subdivided into the conventional CSII (CSII with tubing) and tubeless CSII. The CSII insulin delivery system was then divided into a CSII with an AID system and a CSII with a conventional CSII mode (Manual mode). Finally, it was noted if a patient had a CSII with AID capacity but was using it in a non-AID mode.

### 2.5 Statistical analysis

The normality of continuous variables was assessed by the Kolmogorov-Smirnov test. The characteristics of CSII-starters were compared to MDI-retainers. Normally distributed variables were compared with the student’s t-test. Skewed variables were compared with Wilcoxon-Mann-Whitney test, and categorical variables were compared with chi-square or Fisher exact where appropriate. In paired sub-analysis for the third aim, paired t-test was performed for normally distributed variables and Wilcoxon signed-rank test performed for skewed data. All reported p-values are two-tailed with an alpha of <0.05. Analysis was performed in SAS^®^ 9.4 software (SAS Institute, Cary, NC).

## 3 Results

### 3.1 CSII initiation

A total of 283 individuals met inclusion criteria for aims one and two evaluating CSII start and CSII selection. A slight majority (n=148, 52.3%) were female, and the majority (n=208, 73.5%) were NHW and English-speaking (n=277, 97.9%). A slight minority (n=138, 48.8%), had private insurance. Average age at diabetes diagnosis and pre-pump attendance were 8.8 ± 4.1 years and 11.4 ± 4.1 years respectively.

Among attendees, 280 (98.9%) had a diagnosis of T1D, and 3 (1.1%) had diabetes other than T1D including type 2 diabetes and diabetes due to pancreatic insufficiency. Only two individuals had previously used a CSII; the rest were new to CSII diabetes technology. Most patients used CGM, with a small minority (N = 14, 4.9%) using glucometers. Glucometer users were majority NHB (n = 8; 57.1%), Medicaid insured (n = 12; 85.7%) and female (n = 8; 57.1%). Of the 283 patients who attended pre-pump class (n = 187; 66.1%) were CSII-starters. For CSII-starters, the median time to initiate a CSII after pre-pump attendance was 108 days with an interquartile range of 76–154 days.

CSII-starters and MDI-retainers did not differ in sex, language, nor age at diabetes diagnosis. However, they did differ by race (p=0.0385), and insurance (p=0.0001). Additionally, CSII-starters were younger (p=0.0150), had diabetes for a shorter duration (p=0.0001), lower HbA1c concentrations (p=0.0020), and higher CGM use (p <0.0001) relative to MDI-retainers. (See **Table 1**).

**Table 1:** Demographic information and diabetes-specific data.

	Total (n=283)	CSII starters (n=187)	MDI-retainers (n=96)	*p-value*
Sex: Female (%)	148 (52.3)	99 (52.9)	49 (51.0)	
Race: NHW NHB Hispanic Other	208 (73.5) 58 (20.5) 9 (3.2) 8 (2.8)	147 (78.6) 32 (17.1) 5 (2.7) 3 (1.6)	61 (63.5) 26 (27.1) 4 (4.2) 5 (5.2)	0.0385
Language: Non-English (%)	6 (2.1)	3 (1.6)	3 (3.1)	0.4
Insurance: Private (%)	138 (48.8)	108 (57.8)	30 (31.3)	0.0001
Age at diabetes diagnosis	8.8 (4.1) (n=280)	8.7 (4.0) (n=185)	9.0 (4.2) (n=95)	0.6
Duration of diabetes at pre-pump class^1^	1.13 (0.4-3.7) n=280	0.75 (0.42-2.5) n=185	2.0 (0.75-5.0) n=95	0.0001
Age at Pre-pump class	11.4 (4.1)	11.0 (4.0)	12.2 (4.1)	**0.0150**
Pre-pump HbA1c (%)	8.1 (7.1-9.6)	8.0 (6.9-9.1)	8.6 (7.5-10.4)	0.0020
CGM use at pre-pump	269 (95.0)	187 (100.0)	82 (85.0)	<0.0001

HbA1c, glycated hemoglobin A1c; NHW, non-Hispanic White; NHB, non-Hispanic Black.

Variables are mean (standard deviation) and n (%) unless otherwise indicated.

^1^Median and interquartile range shown for skewed variable.

### 3.2 CSII selection

Most CSII-starters (n= 131, 70%) selected a tubeless CSII. CSII-starters opted for a CSII with an AID system in 68.4% (n=128) of cases, and 90.6% (n=116) of those patients used the CSII in AID mode. The preferred CSII models were: Omnipod^®^ 5 (38.5%), followed by Tandem Control IQ^®^ (27.8%), Omnipod^®^ DASH (19.8%), Omnipod^®^ UST400 (11.8%), Tandem Basal IQ^®^ (1.6%);, and Medtronic MiniMed™ 770 G (0.5%).

At time of CSII initiation, AID-users were 12.0 ± 3.8 years old while those using a non-AID CSII or a CSII with an AID capacity in non-AID mode were 10.4 ± 4.3 years old (p=0.0123). Relative to non-AID CSII users, AID-users had an older age at diabetes diagnosis (p=0.0151). AID use was not associated with pre-pump HbA1c, duration of diabetes mellitus, insurance, sex, race/ethnicity, or language. (See **Table 2**).

**Table 2:** Automated insulin delivery system compared to non-automated insulin delivery system.

	CSII Starters (n = 187)	CSII used with AID (n = 116)	CSII used without AID (n = 71)	*p-value*
Sex: Female (%)	99 (52.9)	56 (48.3)	43 (60.6)	0.1
Race: NHW NHB Hispanic Other	147 (78.6) 32 (17.1) 5 (2.7) 3 (1.6)	88 (75.9) 23 (19.8) 3 (2.6) 2 (1.7)	59 (83.1) 9 (12.7) 2 (2.8) 1 (1.4)	0.6
Language: Non-English (%)	3 (1.6)	3 (2.6)	0	0. 2
Insurance: Private (%)	108 (57.8)	62 (53.5)	46 (64.8)	0.1
Age at diabetes diagnosis	8.7 (4.0) (n=185)	9.3 (3.9) n=115	7.8 (4.1) N=70	
Duration of diabetes at pre-pump class^1^	0.75 (0.42-2.5) n=185	0.67 (0.33-2.75) n=115	0.96 (0.50-2.33) n=70	
Age at CSII start	11.4 (4.0)	12.0 (3.8)	10.4 (4.3)	0.0123
Days to CSII initiation^1^	108 (76-154)	110 (78-162)	102 (71-153)	0.1
Pre-pump HbA1c (%)	8.3 (+/- 1.9)	8.0 (6.9-9.1)	8.1 (7.3-9.0)	0.1

CSII, continuous subcutaneous insulin infusion; AID, Automated insulin delivery system; HbA1C, glycated hemoglobin A1c; NHW, non-Hispanic White; NHB, non-Hispanic Black.

Variables are mean (standard deviation) and n (%) unless otherwise indicated.

^1^Median and interquartile range shown for skewed variable.

### 3.3 Glycemic outcomes

For the glycemic outcome analysis, of the 187 patients that were started on CSII, 183 met the inclusion criteria. One was excluded for not having T1D, one was excluded due to lack of any available CGM data, and two excluded because they were already using a CSII and attended pre-pump class to switch CSII model.

All but 20 patients had a baseline HbA1c measurement. For the 20 subjects without a baseline HbA1c, 14-day CGM data was collected for days 130–144 after CSII initiation, in line with the median time to HbA1c measurement of 144 days for the cohort with HbA1c measurements available (see methods above).

A total of 113 patients were transitioned from MDI to CSII with an AID and 70 were transitioned to a non-AID CSII. Baseline glycemic data, between AID and users of non-AID systems did not differ. However, significant differences were observed between post-CSII glycemic metrics in users of AID systems and users of non-AID systems. The percentage of CGM active time was higher in AID users as compared to users of non-AID systems. Additional changes were noted in mean glucose, glucose management index, time very high, time high, and time in range. No difference was seen in post-CSII time low or very low between users of AID systems and users of non-AID systems (See **Table 3**).

**Table 3:** Glycemic data of CSII starters with T1D.

	All patients n=183	AID users n=113	Users of non-AID systems n=70	p-value
Hemoglobin A1c				
Pre-HbA1c (%)^1^	8.0 (6.9-9.1) n=183	7.7 (6.7-9.1) n=113	7.9 (7.1-8.5) n=70	0.10
Post-HbA1c (%)^1^	7.5 (6.7-8.3) n=163	7.0 (6.5-8.1) n=95	7.9 (7.1-8.5) n=68	0.0021
Pre-CSII CGM Data	n=132	n=87	n=45	
Mean Blood Glucose (mg/dL)	206 (51)	205 (52)	207 (49)	0.77
GMI (%)	8.2 (1.2)	8.2 (1.3)	8.3 (1.8)	0.75
CV (%)	34.9 (6.7)	34.7 (6.6)	35.1 (7.1)	0.78
Time very high^1^ (%)	25.0 (12.0-44.5)	25.0 (11.0-44.0)	24.0 (13.0-46.0)	0.74
Time high (%)	25.3 (9.6)	24.7 (9.6)	26.4 (9.6)	0.33
Time in range^1^ (%)	44.0 (27.5-58.5)	45.0 (28.0-60.0)	43.0 (24.0-54.0)	0.49
Time low^1^ (%)	0 (0.0-1.0)	0 (0.0-1.0)	0 (0.0-1.0)	0.99
Time very low^1^ (%)	0 (0.0-0.0)	0 (0.0-0.0)	0 (0.0-0.0)	0.06
Time CGM active^1^ (%)	97.0 (91.4-98.7)	97.3 (89.2-98.9)	96.9 (93.6-98.4)	0.77
Post-CSII CGM Data	n=142	n=89	n=53	
Mean Blood Glucose (mg/dL)	192 (42)	186 (44)	203 (34)	0.0189
GMI (%)	7.9 (1.0)	7.8 (1.1)	8.25 (0.8)	0.0181
CV (%)	36.3 (6.4)	35.8 (6.5)	37.1 (6.4)	0.23
Time very high (%)	18.5 (10.0-31.0)	15.0 (7.0-27.0)	26.0 (17.0-33.0)	0.0012
Time high (%)	25.0 (10.0)	22.6 (7.1)	29 (12.7)	0.0012*
Time in range (%)	53.0 (39.0-66.0)	61.0 (43.0-70.0)	44.0 (35.0-53.0)	<0.0001
Time low (%)	1.0 (0.0-1.1)	1.0 (0.0-1.0)	0.0 (0.0-1.0)	1.0
Time very low (%)	0 (0.0-0.0)	0 (0.0-0.0)	0 (0.0-0.0)	0.85
Time CGM active (%)	97.9 (95.6-98.8)	98.3 (96.2-98.9)	97.0 (93.5-98.6)	0.0262

HbA1c, glycated hemoglobin A1c; GMI, glucose management indicator; CSII, Continuous subcutaneous insulin infusion; CV, coefficient of variation; CGM, continuous glucose monitor. Data are mean (standard deviation) if normal and median (interquartile range) if skewed. Test for paired skewed data is Wilcoxon signed-rank test. Test for paired normal test is paired t-test.

^1^ Skewed data; * Unequal variances t-test.

Sixty-six patients with T1D for greater than 12 months, transitioned from MDI to CSII, with pre and post CGM data available were included in the sub-analysis. Of these 66, 24 transitioned to non-AID and 42 transitioned to AID. Users of non-AID systems saw no significant changes in pre- and post-glycemic metrics. Specifically, HbA1c went from 7.6% (IQR: 7.0 – 8.7) to 7.6% (IQR: 7.1 – 8.20) and time in range (TIR) went from 44.5% (IQR: 24.5-53.5) to 43.5% (IQR: 30.0-52.5). (See **Supplementary Table 1**).

The cohort of 42 patients who transitioned from MDI to CSII with AID did have glycemic changes. HbA1c went from 8.2% (IQR: 7.4 – 9.4) to 6.9% (IQR: 6.6 – 8.20) (p = <0.0001), and TIR went from 32% (IQR: 22.0-50.0) to 56.5% (IQR: 43.0-66.0) (p = <0.0001). With the exception of *time low, time very low* and *coefficient of variation*, all other CGM metrics differed with AID users having superior outcomes after CSII initiation (See **Supplementary Table 2**).

## 4 Discussion

### 4.1 CSII initiation and selection

Navigating CSII initiation is complex, and many systems struggle to facilitate CSII initiation despite official standards of care recommendations. For many patients at Children’s of Alabama, including all patients insured through Alabama Medicaid, one of the first steps is attending pre-pump class. In this review of 18-months of pre-pump attendees, we found a CSII start rate of 66.1%. Detailed review of the data showed enlightening information about CSII-starters and MDI-retainers.

Before discussing, it is important to highlight this work’s limitations. First, this is a single-center review and results may not generalize to other areas, particularly those with different insurance requirements. Second, the pre-pump class has a capacity of 6 families per class and it is offered 4 times per month. This barrier may explain the time gap between the diagnosis and the pre-pump class date. Third, Alabama Medicaid does not cover CSII until six months post diagnosis, which impacts our duration of diabetes at pre-pump class and may contribute to lower initiation rates. Finally, only those starting CSII by December 2023 were included. With a median time from pre-pump class to CSII initiation of 108 days, it is possible that some patients, particularly those attending pre-pump class towards the end of June 2023, initiated a CSII after December 2023. The CSII start was determined in one of two ways. First, by reviewing the attendance to the CSII start appointment at Children’s of Alabama (CSII initiation date). Second, by reviewing the provider’s documentation at the follow-up clinic visits. As some patients receive CSII initiation directly through the CSII company and not through Children’s of Alabama, if the provider did not accurately document the insulin administration at follow-up, those patients would have been mistakenly identified as MDI-retainers. Finally, available AID systems continued to evolve with changes in availability and coverage during our study period, which might have impacted our results.

Despite these limitations, this work identifies some meaningful insights. In our clinic, approximately 48% of patients with T1D have Medicaid. Although public and privately insured patients were near-equally represented in pre-pump attendance (See **Table 1**), privately insured patients made up a disproportionate number of CSII-starters. In fact, based upon our data showing that for those who attend pre-pump class 78.3% of those with private insurance proceed to CSII start compared to only 54.4% of patients with public insurance. In our population, those with private insurance are 1.4 times more likely to start a CSII than those with public insurance. *Consequently, the type of insurance seems to play a vital role in our population as most MDI-retainers were insured through Alabama Medicaid.*

Similar differences were seen by racial and ethnic group. In our clinic, approximately 30% of patients with T1D are NHB (16), however patients who are NHB made up only 20.5% of pre-pump attendees. For pre-pump attendees who are NHW, 70.1% of them started a CSII compared to only 55.2% of those who are NHB and 55.6% of those who are Hispanic. NHW pre-pump attendees are 1.3 times more likely to start a CSII than NHB and Hispanic pre-pump attendees. This shows disparities in not just CSII initiation, but also access to pre-pump class given the underrepresentation of patients who are NHB in pre-pump class attendees compared to the clinic population.

Although the pre-pump class is available to all patients with diabetes who are interested in starting a CSII, the reason for the underrepresentation of NHB patients in class attendance remains unclear. While detailed information regarding caregivers’ work schedules or primary modes of transportation is lacking, these factors may contribute to the limited participation observed in this population. To enhance accessibility, proposed strategies include offering pre-pump classes at varied times—such as both morning and afternoon sessions—and proactively evaluating the need for transportation support. Additionally, the majority of NHB patients are covered by Medicaid. Therefore, a key area for improvement is reinforcing the insurance requirement mandating attendance at a pre-pump class prior to CSII initiation. Clear communication of this requirement may increase awareness among patients and caregivers, encouraging greater engagement and commitment to attending scheduled sessions. Novel approaches with remote learning should be evaluated given success in other systems (17).

The association of HbA1c with CSII start requires further evaluation. Historically many providers hesitated to initiate CSII in patients with elevated glucose levels. As recently as 2009, expert consensus agreed that suboptimal adherence to diabetes treatment was a relative contraindication to the initiation of a CSII (18). Over the last decades however, practice has changed with the American Diabetes Association’s most recent recommendations stating that CSII therapy should be offered in all patients “who are capable of using the device safely (either by themselves or with a caregiver)” (11). Our institution, has adapted to these guidelines. While previously there was concern about initiating CSII in those with elevated glucose levels due to fears related to increased hospitalization, recent practice has evolved, and an elevated A1C and history of diabetes-related hospitalizations are no longer seen as contraindications to CSII use. Currently, CSII information is routinely offered to all appropriate patients at their first outpatient endocrinology visit. In patients with Medicaid, this conversation may be delayed as Alabama Medicaid will not cover CSII until the patient has had diabetes for a minimum of 6 months.

Interestingly, while our local practice is more inclusive in offering CSII to more patients, our data clearly shows a trend towards lower HbA1c in CSII-starters versus MDI-retainers. It is possible that factors that make it difficult to reach glycemic targets also make it difficult to navigate the complex CSII initiation process (See **Figure 1**). We suspect that it is these outside factors, including high local rates of poverty and food insecurity (19), rather than glycemic management itself, which is the underlying cause of the difference in CSII initiation by HbA1c.

Reassuringly, we found no difference in CSII initiation by language. Even though only 2% of the individuals who attended a pre-pump class were non-English-speaking patients, 50% started a CSII. This finding is reassuring as it is *hard* to get a CSII, more so when operating through a second language (See **Figure 1**). While only 2% of pre-pump attendees were non-English speakers, this is consistent with our patient population with type 1 diabetes, indicating that the non-English speaking patients have access to pre-pump classes at a similar rate as their English-speaking peers.

Findings of age and AID use require a more nuanced evaluation. We saw a slight trend towards lower age in CSII-starters overall but a slightly *higher* age in AID users relative to users of non-AID systems. This likely reflects the preferred CSII model amongst our patients, 70% of whom selected a tubeless model. For a portion of our study period, the tubeless-AID model was not yet available on the formulary for our patients. Our first tubeless AID CSII initiation occurred in the fall of 2022, well into our study period. As AID use becomes approved in lower age ranges with tubeless options, we expect to see a reduction in the age discrepancy amongst CSII users who select AID and non-AID systems. During the study period, while most patients selected a CSII with AID capacity, some used it in non-AID mode as they were using a CGM incompatible with their AID system. Encouragingly, the selection between AID and non-AID did not appear to be influenced by disparities in insurance or race/ethnicity.

Even though the time to start a CSII is somewhat prolonged, this delay may stem from several factors, including communication between the family, CSII company, and insurance; coordination between the CSII company, insurance, endocrine office, and durable medical equipment (DME) provider; Medicaid’s requirement for blood glucose logs; the shipment of CSII equipment; and the CSII training (See **Figure 1**). Advocacy efforts to ensure insurance requirements do not perpetuate disparities to standard-of-care technology are essential.

CSII starters and MDI-retainers did not differ in sex or language but did differ by HbA1c, race/ethnicity, age, duration of diabetes, and insurance. This opens the door for equity-focused improvements and efforts. We look forward to partnering with providers, payors, and families to increase access and use of standard of care technology.

### 4.2 Glycemic outcomes

Our data supports historic data showing superiority of CSII over MDI therapy (15, 20) and adds to it by highlighting clear differences in outcomes for youth not affected by honeymoon period using AID and non-AID CSII systems (See **Supplementary Tables 1**, **2**). Our findings support current standards-of-care with AID as preferred system for youth with T1D (11) with clear differences in outcomes of both HbA1c and CGM glycemic metrics.

### 4.3 Final conclusions

CSII is associated with lower average glucose and better glycemic control (6–8) with AID systems outperforming non-AID systems in our cohort. Unfortunately our data shows relatively low CSII initiation with disparities noted by race, insurance, and glycemic control. When we have a technology available, when used correctly, is associated with improved outcomes, we have an obligation to evaluate and modify our systems to ensure equitable access and use. This work identifies existing disparities in CSII-initiation and provides a baseline to address these barriers for further improvement efforts.

## Data Availability

The raw data supporting the conclusions of this article will be made available by the authors, without undue reservation.
