# Supplementary material for: Patient outcomes after attending pre-pump education class: disparities in initiation and glycemic outcomes
Source: Front Endocrinol (Lausanne). 2025 Jun 5;16:1568133. doi: 10.3389/fendo.2025.1568133 (PMC12176577; doi:10.3389/fendo.2025.1568133)
Supplement: Supplementary file 1 [file Table1.docx]

Supplementary Material

**Supplemental Table 1:** CSII starters with T1D for more than 1 year at the time of Non-AID CSII initiation. Pre/post HbA1c and CGM metrics.

|  | **Before**  **n=24** | **After**  **n=24** | **p-value** |
| --- | --- | --- | --- |
| HbA1c^1^ (%) | 7.6 (7.0-8.7) | 7.6 (7.1-8.2) | 0.24 |
| **CGM Metrics** |  |  |  |
| Mean Blood Glucose (mg/dL) | 210 (51) | 207 (36) | 0.73 |
| CV (%) | 36.6 (6.8) | 37.9 (5.4) | 0.28 |
| GMI (%) | 8.3 (1.2) | 8.3 (0.9) | 0.78 |
| Time very high^1^ (%) | 25.5 (14.0-51.0) | 27.5 (18.0-42.5) | 0.64 |
| Time high (%) | 25.8 (8.1) | 26.5 (7.3) | 0.73 |
| Time in range^1^ (%) | 44.5 (24.5-53.5) | 43.5 (30.0-52.5) | 0.55 |
| Time low^1^ (%) | 1.0 (0.0-2.0) | 1.0 (0.0-1.0) | 0.13 |
| Time very low^1^ (%) | 0.0 (0.0-0.0) | 0.0 (0.0-0.0) | 0.13 |
| Time CGM active^1^ (%) | 96.9 (89.0-98.6) | 96.8 (93.4-98.4) | 0.99 |

*HbA1c: glycated hemoglobin A1c; CV: coefficient of variation; GMI: glucose management indicator; CGM: continuous glucose monitor. Data are mean (standard deviation) if normal and median (interquartile range) if skewed. Test for paired skewed data is Wilcoxon signed-rank test. Test for paired normal test is paired t-test.*

*^1^ Skewed data*

**Supplemental Table 2:** CSII starters with T1D for more than 1 year at the time of AID CSII initiation. Pre/post HbA1c and CGM metrics.

|  |  | **Before**  **n=42** | **After**  **n=42** | **p-value** |
| --- | --- | --- | --- | --- |
| HbA1c^1^ (%) |  | 8.2 (7.4-9.4)  n=42 | 6.9 (6.6-8.2)  n=34 | **<0.0001** |
| **CGM Metrics** |  |  |  |  |
| Mean Blood Glucose (mg/dL) |  | 226 (48) | 197 (51) | **0.0004** |
| CV (%) |  | 35.2 (6.3) | 37.7 (7.1) | 0.9 |
| GMI (%) |  | 8.7 (1.2) | 8.0 (1.2) | **0.0004** |
| Time very high^1^ (%) |  | 36.0 (21.0-53.0) | 17.0 (12.0-30.0) | **<0.0001** |
| Time high (%) |  | 26.3 (7.9) | 22.5 (6.8) | **0.0061** |
| Time in range^1^ (%) |  | 32.0 (22.0-50.0) | 56.5 (43.0-66.0) | **<0.0001** |
| Time low^1^ (%) |  | 0.0 (0.0-1.0) | 1.0 (0.0-1.0) | 0.92 |
| Time very low^1^ (%) |  | 0.0 (0.0-0.0) | 0.0 (0.0-0.0) | 0.44 |
| Time CGM active^1^ (%) |  | 95.5 (87.6-98.2) | 97.7 (95.5-98.9) | **0.0214** |

*HbA1c: glycated hemoglobin A1c; CV: coefficient of variation; GMI: glucose management indicator; CGM: continuous glucose monitor. Data are mean (standard deviation) if normal and median (interquartile range) if skewed. Test for paired skewed data is Wilcoxon signed-rank test. Test for paired normal test is paired t-test.*

*^1^ Skewed data*
